# Lessons learned from COVID-19: Harnessing community insights for better vaccination outcomes

**DOI:** 10.14745/ccdr.v50i10a01

**Published:** 2024-10-03

**Authors:** Theresa Tam

**Affiliations:** 1 Chief Public Health Officer, Public Health Agency of Canada, Ottawa, ON

**Keywords:** COVID-19, preparedness and response, trusted relationships, community approaches, partnerships and innovations

## Introduction

Vaccination is a cornerstone of public health. The COVID-19 pandemic underscored the importance of local, innovative and equity-oriented approaches to achieve comprehensive vaccination coverage, particularly for populations with complex needs. Community leaders and organizations are uniquely positioned to inform and drive efforts that reduce barriers to access and foster supportive environments. They also play a vital role in the vaccination evidence system by supporting the development of research tools and frameworks that resonate with community needs.

Communities are diverse and intersecting by nature. Individuals may belong to multiple groups with shared geography, interests, lived experiences, cultures or identities. Taking a community-oriented approach to vaccination requires us to build strong relationships within and across communities. These relationships can be leveraged through different levels of engagement, from consulting community leaders on existing vaccination programs to supporting community-led projects.

Existing community-based vaccination initiatives ((1)) can offer valuable insights for public health planning of routine and pandemic vaccination programs. Established in 2016, the Public Health Agency of Canada’s Immunization Partnership Fund (IPF) is an example of an initiative that pivoted during the pandemic to be community-oriented ((2)). The IPF funded over 100 community-driven COVID-19 vaccine projects aimed at increasing healthcare provider capacity, supporting community-based education and access initiatives and building capacity for evidence-based communication. Given the success of the projects, the IPF has since expanded its community-oriented approach towards routine and respiratory vaccine projects.

## Leveraging trusted relationships

We achieve better vaccination outcomes when we support trusted community organizations. The following IPF projects used multifaceted approaches that built on pre-existing programs to offer services that prioritized community needs, trusted relationships and transparency.

### Inner City Health Associates

Based in Toronto, Inner City Health Associates (ICHA) employed community health workers (CHWs) with lived experience to facilitate vaccination for individuals experiencing homelessness. These CHWs used destigmatizing approaches to build rapport, foster non-judgemental health discussions, identify high demand clinic locations and adjust scheduling for accessibility. This resulted in 122 pop-up clinics and the vaccination of 1,929 individuals with complex needs from 2023 to 2024.

Inner City Health Associates also relied on community peer ambassadors to co-develop resources, provide training and conduct tailored on-site outreach. Those with medical experience were trained in COVID-19 vaccine administration, enabling peer-led clinics that bridged healthcare and shelter services. Inner City Health Associates are now repurposing these interventions to increase routine and high-risk vaccination services for people experiencing homelessness in Toronto.

### Dr. Peter Centre

Between 2021 and 2024, the Dr. Peter Centre (DPC) administered 37 low barrier microgrants to promote vaccination among underserved populations across Canada. Many recipient organizations described the microgrant model as a “game changer” for hyper-local groups experiencing capacity constraints.

From 2023 to 2024, micrograntees organized 42 tailored vaccination clinics, administering over 2,100 vaccinations in accessible and familiar spaces. Micrograntees addressed intersecting health needs through wrap-around approaches informed by harm reduction models, such as offering COVID-19 vaccinations alongside testing and treatment for sexually transmitted and bloodborne infections. Over the next two years, DPC will continue to address access barriers and empower clients with the knowledge needed to make informed decisions about respiratory and other vaccines.

## Tailored and innovative community approaches

Community engagement can facilitate our understanding of how health information is accessed and used. Many IPF projects leveraged trusted community leaders to act as community ambassadors and to deliver tailored services that helped to create a supportive vaccination environment.

### Alberta International Medical Graduates Association

The Alberta International Medical Graduates Association (AIMGA) used international medical graduates as vaccine navigators during the COVID-19 pandemic to promote vaccination among newcomers in Calgary. These community leaders applied their medical expertise and cultural knowledge to build trust and reduce language barriers for vaccination services. By partnering with Alberta Health Services and other immigrant-serving organizations, they were also instrumental in providing thousands of COVID-19 vaccines to at-risk workers at meat processing facilities in Alberta.

From 2023 to 2024, AIMGA continued to enhance access to evidence-based health information for diverse populations through culturally safe, multi-lingual clinics and social media campaigns in 25 languages. Building on their pandemic efforts, AIMGA is now working to increase vaccine literacy and uptake of routine and respiratory virus vaccines in Calgary’s newcomer populations.

### Regroupement des centres d’amitié autochtones du Québec

*Regroupement des centres d’amitié autochtones du Québec* (RCAAQ), a collective providing culturally safe services to urban Indigenous populations in Québec, led the Miro Matisiwin project (“Wellbeing”) during the pandemic. This initiative used fixed-site and mobile clinics to increase COVID-19 vaccine uptake among individuals who were not well-served by traditional vaccination services.

Expanding on the learnings of Miro Matisiwin, RCAAQ launched the Mamu project (“Together”) from 2023 to 2024 to enhance awareness and uptake of routine and seasonal vaccinations. As part of this program, RCAAQ developed culturally relevant promotional materials to reach 120,000 online users. *Regroupement des centres d’amitié autochtones du Québec* continues to promote routine and seasonal vaccinations in urban Indigenous populations by prioritizing culturally safe and trauma-informed care, decreasing access barriers to vaccination and developing tailored resources.

## Conclusion

Now is the opportune time to reflect on how we can sustain the community partnerships and innovations developed prior to, during and post the COVID-19 pandemic. As I raised in my 2023 report, this will require us to consistently integrate community-centred planning across our preparedness and response efforts, including broader pandemic planning, outbreak responses and routine programming ((3)). We can support the full participation of communities in these efforts through streamlined and coordinated funding mechanisms that meet the needs of community organizations. If we are intentional in our efforts to fortify community relationships and be inclusive of community perspectives, we will be working towards a future in which everyone can experience the benefits of vaccination and, ultimately, better health outcomes against vaccine-preventable diseases.
